# Exploring the Protective Efficacy of Topical Products for Actinic Keratosis Against Ultraviolet-Induced DNA and Protein Damage: An Experimental, Double-Blind Irradiation Study

**DOI:** 10.7759/cureus.44065

**Published:** 2023-08-24

**Authors:** Piercarlo Minoretti, Enzo Emanuele, Ángel García Martín, Miryam Liaño Riera, Manuel Gómez Serrano, Andrés Santiago Sáez

**Affiliations:** 1 General Direction, Studio Minoretti, Oggiono, ITA; 2 Scientific Directorate, 2E Science, Robbio, ITA; 3 Legal Medicine, Psychiatry and Pathology, Complutense University of Madrid, Madrid, ESP; 4 Legal Medicine, Hospital Clinico San Carlos, Madrid, ESP; 5 Legal Medicine, Psychiatry, and Pathology, Complutense University of Madrid, Madrid, ESP

**Keywords:** protein carbonylation, cyclobutane pyrimidine dimers, dna, ultraviolet radiation, actinic keratosis

## Abstract

Introduction

Numerous studies have delved into the clinical efficacy of different topical treatments for actinic keratosis (AK). However, our understanding remains limited regarding their capacity to prevent DNA and protein damage caused by ultraviolet radiation (UVR).

Objectives

The aim of this study was to analyze and compare the preventive capabilities of various AK-targeted products in countering DNA and protein alterations in human biopsies following exposure to experimental UVR.

Methods

Twelve healthy Caucasian volunteers (six men and six women) aged 18 years and above, with Fitzpatrick skin types II−III, participated in an experimental irradiation study. Six topical products, containing various ingredients (DNA repair enzymes, antioxidants, keratolytic agents, cyclooxygenase inhibitors, and/or sunscreens) were tested. The experimental sites were exposed to UVR at six times the minimal erythema dose for eight consecutive days. Each test product was applied 30 to 45 minutes before irradiation at a standard thickness of 2 mg/cm^2^. A control site was treated with the vehicle alone, serving as a negative control. The study focused on cyclobutane pyrimidine dimers (CPDs) and protein carbonylation (PC) as molecular markers of UVR-induced DNA and protein damage, respectively.

Results

The efficacy of different AK-targeted topical products showed substantial variation when applied to normal skin before experimental exposure to UVR. While sunscreens, predictably, played a crucial role, additional ingredients (i.e., DNA repair enzymes and antioxidants) also acted as vital protective agents for both the cellular genome and proteome, shielding them against UVR-induced damage.

Conclusion

In topical products specifically designed for AK, the strategic integration of DNA repair enzymes and antioxidants, in addition to sunscreens, establishes a critical defense mechanism against the detrimental effects of UVR on cellular DNA and proteins.

## Introduction

Actinic keratoses (AKs) are enduring and frequently recurring skin abnormalities characterized by their keratotic nature, variable thickness, and frequently indistinct borders [1,2]. These lesions primarily develop on areas of the skin that have been consistently exposed to ultraviolet radiation (UVR). Although some AKs may regress, they usually persist and have the potential to evolve into squamous cell carcinoma (SCC), making them precancerous in nature [3]. However, malignant skin lesions may also originate from clinically unremarkable, sun-damaged areas surrounding AKs, a phenomenon commonly referred to as field cancerization (FC) [4]. Essentially, FC signifies the persistent existence of molecular abnormalities that can potentially drive cutaneous cancerogenesis over time [5].

Within the UVR spectrum, the UVB wavelength, specifically ranging from 280-320 nm, has the unique capability to directly excite the nucleotides present in DNA [6]. This in turn results in the direct formation of specific photoproducts known as cyclobutane pyrimidine dimers (CPDs). Notably, CPDs are believed to play a crucial role in the initiation and accumulation of mutations associated with precancerous and cancerous skin lesions [7,8]. In addition to fostering the formation of CPDs, UVR, especially the UVA wavelength (320-400 nm), can also trigger the generation of reactive oxygen species (ROS), which may in turn cause indirect, oxidative-induced molecular damage [9]. In general, the skin possesses a range of antioxidant mechanisms that serve to safeguard it against oxidative damage caused by ROS. These mechanisms can be categorized into enzymatic (such as copper-zinc superoxide dismutase and catalase) and non-enzymatic (including glutathione) [10]. However, in cases where these defense mechanisms are overwhelmed or insufficient, protein carbonylation (PC) can occur, resulting in oxidative modifications at the protein level. PC not only disrupts the functionality of endogenous DNA repair enzymes due to ROS-induced dysfunction but can also hinder the effective elimination of premalignant cells through apoptosis [9]. Concurrent with other processes implicated in photocarcinogenesis, such as immunosuppression, inflammation, and lipid peroxidation [11], these genomic and proteomic modifications can provide an environment conducive to the establishment, expansion, and progression of transformant clones. Remarkably, a recent study has unveiled a compelling correlation between non-melanoma skin cancer and elevated levels of oxidative damage markers (including PC) [12]. This research also highlighted the pivotal role that vitamin D deficiency plays in an individual's vulnerability to oxidative stress during photocarcinogenesis [12].

In addition to traditional lesion-directed treatments such as cryotherapy, laser therapy, surgery, and curettage, as well as field-directed treatments like topical 5-fluorouracil, diclofenac gel, chemical peeling, imiquimod, and photodynamic therapy [13], there is currently a wide range of topical products available for the prevention and/or adjuvant treatment of AKs and associated FC [13,14]. Based on an improved understanding of AK’s pathophysiology, these formulations contain different mixtures of key ingredients like chemical sunscreens, DNA repair enzymes (e.g., photolyase, endonuclease, and glycosylase), antioxidants, keratolytic agents (e.g., urea), and cyclooxygenase (COX) inhibitors (e.g., diclofenac and piroxicam) [14].

While extensive research has been conducted on the clinical effectiveness of various topical preparations in patients with AKs and related FC [13,14], there is limited knowledge about their ability to prevent the formation of AK-related molecular alterations in human skin biopsies following experimental UVR irradiations. In this experimental double-blind irradiation study, we focused on CPDs and PC as key molecular markers of UVR-induced DNA and protein damage, respectively [8,9]. The research was conducted within a conceptual framework aimed at enhancing the genomic and proteomic integrity of skin cells after repeated UVR exposure, with the ultimate objective of reducing the risk of AK in a personalized and targeted manner.

## Materials and methods

Subjects

This investigation forms an integral component of a comprehensive research endeavor, aimed at evaluating various sun defense products by analyzing biomarkers of UVR-induced DNA and protein damage [15-18]. The ultimate goal is to delve into the molecular level and determine the protective effectiveness of these products, with the aim of developing a personalized approach to UVR protection. The study included twelve healthy Caucasian volunteers (six men and six women; mean age 32.2 ± 4.6 years) aged above 18 years, all possessing a Fitzpatrick skin type II−III who volunteered to partake in an experimental irradiation study. Criteria for exclusion included a history of photodermatosis, skin cancer, or AK, as well as any active or previously treated cancer or malignancy. Individuals on photosensitizing or anti-inflammatory medications were also not considered for the study. Ineligibility extended to those aged under 18 years or over 65 years, and those with a history of inflammatory or atopic disorders. Those suffering from serious non-malignant diseases such as cardiovascular or pulmonary disorders, systemic lupus erythematosus, or scleroderma were also deemed ineligible. Pregnancy, breastfeeding, or psychiatric disorders that could hinder the process of obtaining informed consent were other exclusion factors. This research strictly adhered to the ethical guidelines outlined in the Declaration of Helsinki and was approved by Studio Minoretti(approval number: 2019/07EI). Written informed consent was obtained from all participants.

Test materials

The test materials were composed of a selection of commercial preparations specifically designed for AK. These included 1) Fotoker (Pharcos, Tavarnelle Val di Pesa, Italy), 2) Eryfotona AK-NMSC Fluid (ISDIN, Barcelona, Spain), 3) Heliocare 360 MD AK Fluid (Cantabria Labs Difa Cooper, Caronno Pertusella, Italy), 4) Actixicam (Cantabria Labs Difa Cooper), 5) Kerà K1 (Giuliani, Milan, Italy), and 6) Kerà K2 (Giuliani). A comprehensive summary of the test materials’ general characteristics can be found in Table 1.

**Table 1:** General characteristics of the test materials

Commercial product name	Sun protection factor	Presence of DNA repair enzyme (concentration)	Other key ingredients	Clinical indications and claims
Fotoker	Not declared	Liposomal photolyase (1%)	Diaminopropionoyl tripeptide-33, vitamin E	Treatment for improving actinic keratosis symptoms while proactively aiding in the prevention of DNA damage instigated by UV radiation
Eryfotona AK-NMSC Fluid	100+	Encapsulated photolyase (concentration not declared)	Bisabolol, pantenol, tocopheryl acetate	Treatment for improving field cancerization linked with actinic keratosis and other non-melanoma skin cancer types
Heliocare 360 MD AK Fluid	100+	Photolyase, endonuclease, glycosylase (concentrations not declared)	Polypodium leucotomos extract, green tea, vitamin C, vitamin E, arginine, Vederine® (oligofructosans of chicory)	Proactive prevention and protective coadjuvant treatment for actinic keratosis and other non-melanoma skin cancer types
Actixicam	50+	None	Polyvinyl alcohol, piroxicam	Treatment for actinic keratosis designed to enhance cellular trophism; the product promotes skin hydration and alleviates symptoms of redness and itching
Kerà K1	Does not contain sunscreens	None	Lactic acid, hyaluronic acid, octatrienoic acid	Treatment of actinic keratoses with limited thickness; adjuvant in managing skin aging
Kerà K2	Does not contain sunscreens	None	Urea, lactic acid, octatrienoic acid	Removal of hyperkeratosis by reestablishing the physiological mechanisms of keratinization; promotion of cellular renewal

The study utilized a common moisturizer base as the negative control, and all test materials were anonymized to avoid bias. To ensure objectivity, both participants and personnel were blinded to the product applied prior to experimental irradiations.

Ultraviolet radiation source

The Oriel Solar Simulator (Model 81292, L.O.T. Oriel, Leatherhead, UK) was used to generate solar-simulated UVR. It employs a 1 kW xenon arc lamp, a pair of dichroic mirrors, a collimator, and a 1-mm WG320 filter. This specific simulator’s optical design ensures a uniform irradiance field (290−400 nm) on the skin surface when placed 11 cm from the source, with approximately 10% being UVB (280−320 nm) and the rest as UVA. Spectral irradiance was gauged using an OL754 spectroradiometer (Optronics, Orlando, FL, USA), calibrated against standard lamps for both wavelength and intensity. The spectroradiometer was then applied to calibrate an IL700 handheld radiometer (International Light, Newburyport, MA, USA), used for monitoring of the lamp’s output.

Irradiation and treatment protocol

Two weeks prior to the test irradiations, the minimal erythema dose (MED) − a measure of the smallest amount of solar-simulated UVR (290−400 nm) that produces visible redness on the skin − was determined for each participant. The irradiated zones were scrutinized 24 hours post-irradiation and the MED was identified at the location that displayed the minimal, yet consistent, uniform perceptible erythema. Prior to the irradiation process, we marked seven circular areas on the unexposed lower back of each participant. Each circle had a diameter of 10 mm and represented one of the six test products, along with a negative control site. For eight consecutive days, these experimental sites were subjected to UVR at six times the MED. Between 30 to 45 minutes prior to each experimental irradiation, we applied each test product onto the circular irradiation areas (10 mm diameter, total area of 78.5 mm2 each) with a standard application thickness of 2 mg/cm2. A designated control site underwent treatment with the vehicle prior to the application of UVR, acting as a negative control in the experiment. All participants were required to report to the study center for all irradiations, with investigators being responsible for all test product applications. A day after the final UVR exposure, skin specimens were collected from all sites using a 4-mm punch biopsy for subsequent molecular analyses.

Laboratory measurements

In keeping with the previous methodology [15-18], the skin biopsy specimens were split into two segments. One part was gently thawed at ambient temperature, finely minced, and then subjected to a cycle of freeze-thawing three times. This process involved freezing in an ethanol-dry ice bath, followed by thawing at an elevated temperature of 95°C. For the purpose of quantifying CPDs, the samples underwent a digestion process lasting 12 hours at a moderate temperature of 60°C. This was performed with proteinase K in a solution containing 100 mmol/liter Tris-HCl (at a pH of 7.4), 150 mmol/L NaCl, and 10 mmol/L EDTA (with a pH of 8.0). Subsequently, proteinase K was deactivated by heat treatment at 95°C for a duration of 10 minutes. The homogenates were then extracted using the comprehensive Puregene DNA isolation kit (Gentra Systems, Minneapolis, MN, USA). This kit encompasses two primary reagents: cell lysis and protein precipitation solutions. In brief, DNA was extracted from the homogenates utilizing a lysis buffer solution, followed by an RNase A treatment. The kit employs a protein precipitation solution to remove proteins, which is followed by the addition of 2-propanol to form a DNA pellet. CPDs were quantified in a duplicate manner using a specific ELISA kit (OxiSelect Cellular UV-Induced DNA Damage ELISA Kit, CPDs; Cell Biolabs, San Diego, CA, USA) according to the manufacturer’s guidelines. PC in cell lysates was measured with the OxiSelect™ Protein Carbonyl ELISA Kit (Cell Biolabs). The results obtained from CPDs and PC these measurements were reported in arbitrary units that represent the relative absorbance in ELISA compared to an untreated control (which was established as 1 by convention). All laboratory analyses were performed in a blinded manner, without knowledge of the irradiation protocol.

Data analysis

One-way analysis of variance was used to determine intergroup differences in CPDs and PC, followed by Newman-Keuls multiple-comparison post hoc tests. We did not apply a Bonferroni correction due to the exploratory nature of the study. Statistical analyses were performed using GraphPad Prism 7.0 (GraphPad Inc., San Diego, CA, USA). A two-tailed P value of less than 0.05 was considered statistically significant.

## Results

Cyclobutane pyrimidine dimers

**Figure 1:**
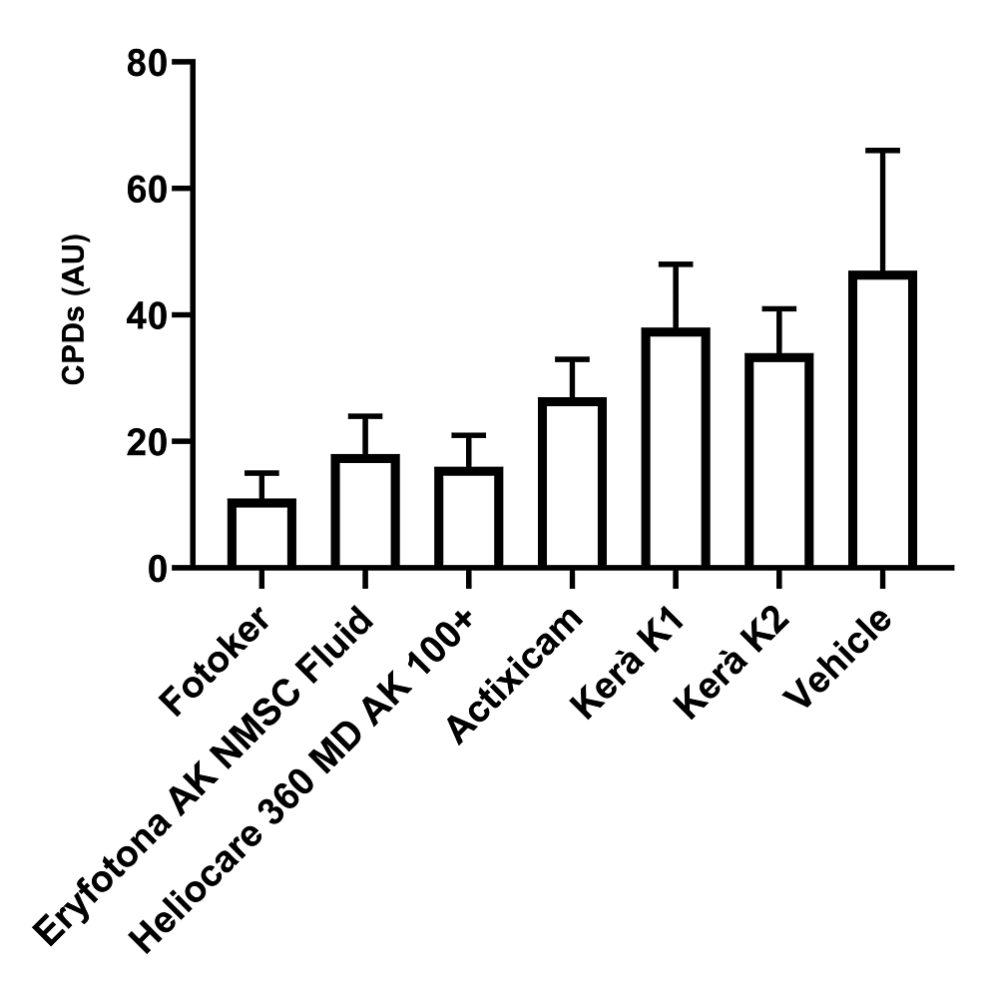
Assessment of the protective influence of diverse topical treatments for actinic keratosis via post-irradiation measurements of CPDs in skin biopsies CPD: cyclobutane pyrimidine dimers

The data presented in Table 2 and Figure 1 demonstrate that Fotoker, which contains liposomal photolyase at a concentration of 1%, outperformed all other test materials in reducing CPDs after experimental irradiations (all P < 0.05). In fact, Fotoker proved to be 31.2% more effective in reducing CPDs compared to the next most effective product, Heliocare 360 MD AK Fluid, which contains photolyase, endonuclease, and glycosylase at unspecified concentrations.

**Table 2:** Protective effect of various topical products for actinic keratosis assessed by measuring CPDs in skin biopsies post-irradiation Results are given as arbitrary units CPD: cyclobutane pyrimidine dimers

Product	Mean	Standard deviation
Fotoker	11	4
Eryfotona AK-NMSC Fluid	18	6
Heliocare 360 MD AK Fluid	16	5
Actixicam	27	6
Kerà K1	38	10
Kerà K2	34	7
Vehicle	47	19

Protein carbonylation

**Figure 2:**
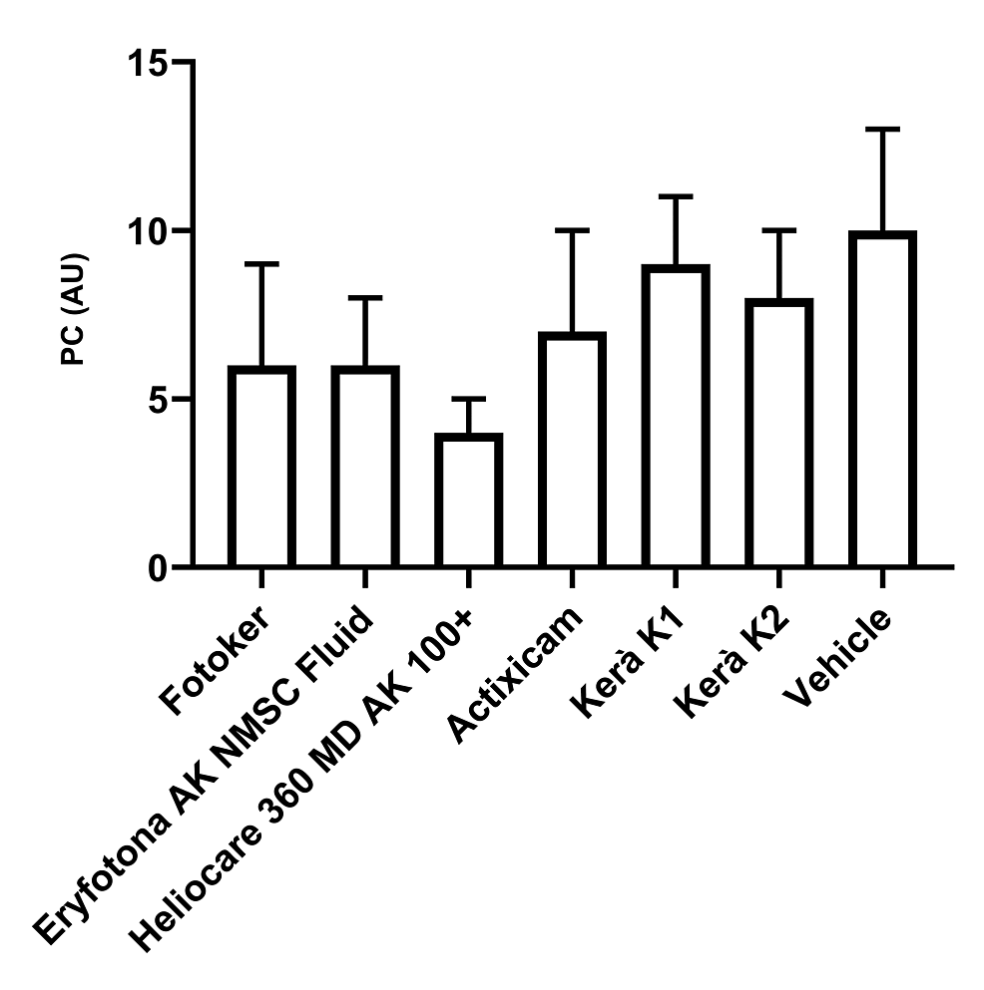
Assessment of the protective influence of diverse topical treatments for actinic keratosis via post-irradiation measurements of PC in skin biopsies PC: protein carbonylation

The data presented in Table 3 and Figure 2 indicate that Heliocare 360 MD AK Fluid outperformed all other tested materials in reducing post-irradiation PC (all P < 0.05). Notably, when compared to the next most potent products, Eryfotona AK-NMSC Fluid and Fotoker, the efficacy of Heliocare 360 MD AK Fluid in reducing PC post-irradiation was superior by 33.3%.

**Table 3:** Protective effect of various topical products for actinic keratosis assessed by measuring PC in skin biopsies post-irradiation Results are given as arbitrary units PC: protein carbonylation

Product	Mean	Standard deviation
Fotoker	6	3
Eryfotona AK-NMSC Fluid	6	2
Heliocare 360 MD AK Fluid	4	1
Actixicam	7	3
Kerà K1	9	2
Kerà K2	8	2
Vehicle	10	3

## Discussion

The findings of our study suggest that the molecular effectiveness of various topical formulations, designed to serve as adjuvant treatment against AK, can display considerable variation when applied to normal skin exposed to experimental UVR. This variability becomes apparent when observing the varying degrees to which they can prevent the formation of skin biomarkers associated with UVR-induced DNA (i.e., CPDs) and protein (i.e., PC) damage. Specifically, our research yielded three key insights that deserve particular attention. Firstly, two of the six tested products (Kerà K1 and Kerà K2) demonstrated no significant molecular protection against damage resulting from experimental irradiations. This lack of significant protective capacity is likely attributable to inadequate sun protection factor (SPF) levels and the absence of specific protective ingredients, such as DNA repair enzymes, potent antioxidants, or anti-inflammatory molecules, that can effectively safeguard the cellular genome and proteome from both direct and indirect (oxidative) UVR-induced damage. Consequently, while products containing urea, lactic acid, hyaluronic acid, and octatrienoic acid may prove beneficial in reducing hyperkeratinization, they are not expected to ameliorate or rectify the molecular damage caused by UVR or contribute to the reversal of FC. Secondly, Fotoker - a topical cream comprising 1% liposomal photolyase - excelled as the most effective topical formulation in preventing the formation of CPDs following experimental UVR exposure on normal human skin. Photolyase, a DNA-repair flavoprotein enzyme, repairs CPDs using blue light as an energy supply [19,20]. When encapsulated in liposomes, photolyase can penetrate the nuclei of keratinocytes, facilitating direct CPD removal [15]. The significant effectiveness of Fotoker, when CPDs were the biomarker of interest, likely stems from the combination of an elevated (although undisclosed) SPF and the relatively high concentration of photolyase. This blend seemingly enhances the product’s capacity to rectify DNA damage directly caused by UVB wavelengths [8]. Thirdly, Heliocare 360 MD AK Fluid demonstrated superior performance over other competitors in reducing protein damage, as indicated by PC measurements. This product boasts an intricate formulation that includes three distinct DNA repair enzymes (photolyase, endonuclease, and glycosylase), and several antioxidants like *Polypodium leucotomos* extract [21], green tea [22], and vitamins C and E [23]. We hypothesize that the remarkable efficacy of this product in reducing PC may be credited to its high antioxidant potential. These antioxidants may mitigate the formation of ROS induced by UVA radiation, thereby subsequently reducing protein oxidation [24]. When contrasted with other assessed products, Eryfotona AK-NMSC Fluid showed a performance similar to Fotoker in terms of PC. However, in the case of CPDs, Eryfotona AK-NMSC Fluid fell significantly short of Fotoker’s effectiveness. This discrepancy may be due to a similar antioxidant efficacy but a lower concentration of photolyase, or possibly a less efficient process for intracellular delivery of the enzyme to the cell nucleus.

The study highlights not only the importance of sunscreens but also draws attention to the critical role that specific ingredients (i.e., DNA repair enzymes and antioxidants) play in shielding both the cellular genome and proteome from the potential direct and indirect damage caused by UVR. As a whole, our results underscore the significance of utilizing AK products that go beyond merely moisturizing and exfoliating. Conversely, these products should also offer protective measures and/or repair mechanisms to mitigate UVR-induced molecular damage. Our data also underscore the crucial role of the DNA-repair flavoprotein enzyme photolyase, a vital player in the direct repair of CPDs triggered by the UVB wavelength in the UVR spectrum [15,19,20]. Furthermore, a comprehensive strategy appears to demonstrate superior efficacy in combating UVR damage. This approach could incorporate DNA repair enzymes for the direct repair of damage induced by UVB, and tackle UVA-induced oxidative protein damage indirectly via antioxidants. However, the effectiveness of the tested formulations varied, depending on the specific skin biomarkers examined. Therefore, additional research is essential to thoroughly understand their protective capabilities and refine their formulations for maximum effectiveness in patients with AK.

Our findings need to be interpreted in the context of several limitations. Primarily, the study’s generalizability was constrained due to the limited number of participants, all of whom only represented two distinct Fitzpatrick skin types (II and III). Secondly, this experimental research did not investigate if the observed variations in CPDs and PC formation between different products have any significant clinical impact on AKs. This particular research question was beyond the scope of our study, presenting a need for further clinical investigations. These future studies should aim to compare the proficiency of various products in lessening the count of existing and new AK lesions, either individually or in conjunction with therapeutic strategies such as photodynamic therapy or cryotherapy [25]. Furthermore, it remains undetermined which product would allow for more extended intervals between photodynamic therapy or cryotherapy sessions for patients with AK. Thirdly, our research design intentionally incorporated similar formulations in the tested products. Within the landscape of AK topical creams, we sought to explore their distinct molecular impacts. This deliberate strategy aimed to expose the subtle distinctions which might not be immediately perceptible due to comparable composition and functionalities. Our goal was to offer a more exhaustive comparison, underscoring the unique traits and contributions of each product against AK. Lastly, the absence of focus on the UVA protection factor is a constraint inherent to our study. Regrettably, we faced difficulties gathering UVA protection data from certain product manufacturers, hindering us from delivering an all-encompassing view of the broad-spectrum sun protection these products offer.

## Conclusions

In topical products specifically designed for AK, the strategic integration of DNA repair enzymes and antioxidants, in addition to sunscreens, establishes a critical defense mechanism against the detrimental effects of UVR on cellular DNA and proteins. By incorporating these ingredients, the effectiveness of these products goes beyond basic moisturizing and exfoliating functionalities, offering enhanced protection against UVR damage.
